# Prognostic Value of High-Sensitivity Troponin in Predicting Long-Term Cardiovascular Outcomes in Patients With Type 2 Diabetes: A Systematic Review

**DOI:** 10.7759/cureus.83925

**Published:** 2025-05-11

**Authors:** Muhammad Hamza Shahid, Nisha Ali, Mounica Ratnala, Rehan Ishaque, Hamesh Gundala Raja, Roohollah Rahbani, Mir Ashar Ali, Mohammed Sadat Mohiuddin, Haroon Bin Idrees, Nishat Jahan Chowdhury, Amir Ali

**Affiliations:** 1 Internal Medicine, Akhtar Saeed Medical and Dental College, Lahore, PAK; 2 Internal Medicine, Liaquat University Hospital, Hyderabad, PAK; 3 Internal Medicine, Anam Chenchu Subba Reddy Government Medical College, Nellore, IND; 4 Peadiatrics, Liaquat University of Medical and Health Sciences, Jamshoro, PAK; 5 Internal Medicine, K.A.P. Viswanatham Government Medical College, Trichirappalli, IND; 6 Internal Medicine, First Moscow State Medical University, Sydney, AUS; 7 Internal Medicine, Deccan College of Medical Sciences, Hyderabad, IND; 8 Internal Medicine, Avicenna Medical College, Lahore, PAK; 9 Internal Medicine, Sylhet Women's Medical College, Sylhet, BGD; 10 Internal Medicine, Services Hospital Lahore, Lahore, PAK

**Keywords:** biomarker, cardiovascular events, high-sensitivity troponin, hs-tni, hs-tnt, long-term outcomes, prognosis, risk stratification, systematic review, type 2 diabetes mellitus

## Abstract

Cardiovascular disease remains the leading cause of morbidity and mortality among patients with type 2 diabetes mellitus (T2DM). While conventional risk assessment tools often lack precision in diabetic populations, high-sensitivity cardiac troponins (hs-cTn), particularly high-sensitivity troponin T (hs-TnT) and high-sensitivity troponin T I (hs-TnI), have emerged as promising biomarkers capable of detecting subclinical myocardial injury and predicting adverse cardiovascular outcomes. This systematic review aimed to evaluate the prognostic effectiveness of hs-cTn in predicting long-term cardiovascular events in individuals with T2DM. A comprehensive search of PubMed, Scopus, and Web of Science was conducted for studies published up to March 2025, limited to English-language human studies. Only four studies met the inclusion criteria, reflecting the limited but focused evidence base. These studies varied in population characteristics, ranging from stable outpatients to patients with diabetic nephropathy and post-acute coronary syndrome, and in the outcomes assessed, including cardiovascular and all-cause mortality, heart failure, and renal events. Given this heterogeneity in both populations and endpoints, a narrative synthesis approach was adopted. Despite differences, elevated hs-cTn levels were consistently associated with worse long-term outcomes, although the strength of associations varied, with more consistent predictive value observed for cardiovascular and all-cause mortality compared to renal events. While findings support the potential of hs-cTn as a risk stratification tool in T2DM, the small number of eligible studies and variability in study designs limit generalizability and highlight the need for further research.

## Introduction and background

Cardiovascular disease (CVD) remains the leading cause of morbidity and mortality in individuals with type 2 diabetes mellitus (T2DM), despite advancements in glycemic control and therapeutic interventions [1]. Patients with T2DM are at a significantly elevated risk of experiencing major adverse cardiovascular events (MACE), including myocardial infarction, stroke, heart failure, and cardiovascular-related death [2,3]. This heightened risk is often attributed to a complex interplay of insulin resistance, chronic inflammation, endothelial dysfunction, and accelerated atherosclerosis [4]. Early identification of high-risk individuals within the diabetic population is therefore crucial for targeted intervention, better prognosis, and optimized resource allocation.

Traditional risk stratification tools such as the Framingham Risk Score [5] or United Kingdom Prospective Diabetes Study (UKPDS) Risk Engine [6], though useful, often fall short of accurately predicting long-term cardiovascular outcomes in diabetic cohorts. This gap in predictive precision has driven interest in novel biomarkers that reflect underlying subclinical myocardial injury. High-sensitivity cardiac troponins (hs-cTn), particularly high-sensitivity troponin T (hs-TnT) and high-sensitivity troponin T I (hs-TnI), have emerged as promising candidates due to their ability to detect minute levels of cardiomyocyte damage even in the absence of clinical symptoms [7]. While initially developed for the early diagnosis of acute coronary syndromes, accumulating evidence from cohort studies and post hoc analyses suggests that elevated hs-cTn levels may have independent prognostic value in predicting long-term cardiovascular outcomes, even among asymptomatic individuals with T2DM.

Despite these promising signals, existing evidence remains heterogeneous in several aspects. Studies have used varying hs-cTn thresholds (e.g., sex-specific cutoffs vs. population-based percentiles), assay types (hs-TnT vs. hs-TnI), and differing follow-up durations ranging from short-term assessments to over a decade. Moreover, the populations studied have included both stable outpatients and higher-risk individuals, such as those with post-acute coronary syndrome (ACS), making it difficult to generalize findings across the broader diabetic population. The lack of uniformity in study designs and inconsistent outcome reporting underscores the need for a structured synthesis of current evidence.

Recent studies have begun to explore the role of hs-cTn as a tool for long-term risk assessment in stable type 2 diabetic patients, particularly in predicting all-cause mortality, cardiovascular mortality, and hospitalization due to cardiac complications. However, clinical integration of this biomarker into standard risk stratification models for diabetics remains limited, in part due to the aforementioned variations across studies. A systematic review of the available literature is therefore warranted to clarify the role of high-sensitivity troponin in forecasting long-term cardiovascular events in this high-risk group and to assess whether predictive value varies by population type or assay used.

This systematic review is structured around the Patient, Intervention, Comparison, Outcome (PICO) framework [8] to ensure clarity and clinical relevance. The population of interest comprises adult patients diagnosed with type 2 diabetes mellitus, irrespective of prior cardiovascular history. Given the variation in study populations (e.g., stable outpatients versus post-ACS patients), this review interprets findings in context and highlights differences when applicable. The intervention or exposure under evaluation is the measurement of high-sensitivity cardiac troponin (hs-cTnT or hs-cTnI) levels, assessed either as a single value or serially, in outpatient or post-ACS settings. The comparator may include patients with lower or undetectable hs-cTn levels, standard risk prediction models without biomarker integration, or alternative biomarkers used for cardiovascular risk prediction. The primary outcomes of interest are long-term cardiovascular events, which include MACE such as myocardial infarction, stroke, hospitalization for heart failure, and cardiovascular mortality. A minimum follow-up duration of six months was chosen to ensure adequate time for meaningful cardiovascular event capture and reduce the risk of short-term biases. This framework aims to evaluate the prognostic value of high-sensitivity troponin testing in predicting adverse cardiovascular outcomes among patients with type 2 diabetes, thus informing its potential utility in clinical practice.

## Review

Materials and methods

Search Strategy

A comprehensive search strategy was employed in accordance with the Preferred Reporting Items for Systematic Reviews and Meta-Analyses (PRISMA) guidelines to ensure transparency and reproducibility in study selection [9]. Electronic databases, including PubMed, Scopus, and Web of Science, were systematically searched for relevant literature published up to 2025. The search strategy combined Medical Subject Headings (MeSH) and free-text terms such as “high-sensitivity troponin,” “type 2 diabetes,” “cardiovascular events,” and “risk prediction.” Filters were applied to include only human studies published in English, with a focus on randomized controlled trials, cohort studies, and biomarker-based analyses. Additional manual screening of reference lists from included articles was performed to capture any potentially relevant studies not retrieved through database searches. All identified articles were independently screened by multiple reviewers, with disagreements resolved by consensus, ensuring a rigorous and unbiased selection process.

Eligibility Criteria

Studies were deemed eligible for inclusion in this systematic review if they investigated adult patients with type 2 diabetes mellitus (T2DM) and evaluated high-sensitivity cardiac troponin (hs-cTnT or hs-cTnI) as a predictor of long-term cardiovascular outcomes. Eligible study designs included randomized controlled trials (RCTs), post hoc analyses of RCTs, and prospective or retrospective cohort studies. Only studies that reported clinical endpoints such as cardiovascular mortality, all-cause mortality, heart failure hospitalization, myocardial infarction, or composite cardiovascular events over a follow-up period of at least six months were included. Articles were limited to those published in English and involving human participants.

Studies were excluded if they focused on type 1 diabetes, acute coronary syndrome management, pediatric populations, or if they did not include hs-cTn as a primary or secondary biomarker. In addition, studies that lacked clear outcome measures, had insufficient data for extraction, or were reviews, editorials, or conference abstracts without complete data were excluded. When duplicate data from overlapping populations were found, the most comprehensive and recent report was retained for analysis.

*Data Extraction* A standardized data extraction form was developed and used to collect key information from each included study. Extracted data included study title, authors, year of publication, study design, population characteristics, sample size, duration of follow-up, type and cutoff levels of hs-cTn used, clinical outcomes assessed, and key findings. The extraction was conducted independently by two reviewers to ensure accuracy, with discrepancies resolved through discussion and consensus. This structured approach ensured consistency and minimized bias in data collection.

*Data Analysis and Synthesis* Given the heterogeneity of study designs, outcome measures, and reporting formats, a narrative synthesis approach was used to summarize findings across the included studies. Studies were grouped based on similarities in population characteristics, biomarker usage, and outcome assessment. Key results were compared qualitatively, highlighting patterns in predictive value, performance metrics (e.g., hazard ratios and C-statistics), and the role of hs-cTn in various clinical contexts. The overall risk of bias was assessed using appropriate tools (Cochrane Risk of Bias (RoB), version 2.0 (www.cochrane.org) for randomized controlled trials (RCTs) and the Newcastle-Ottawa Scale for cohort studies) to evaluate the strength and consistency of the evidence. Quantitative meta-analysis was not performed due to variability in study designs and outcome definitions.

Results

Study Selection Process

**Figure 1:**
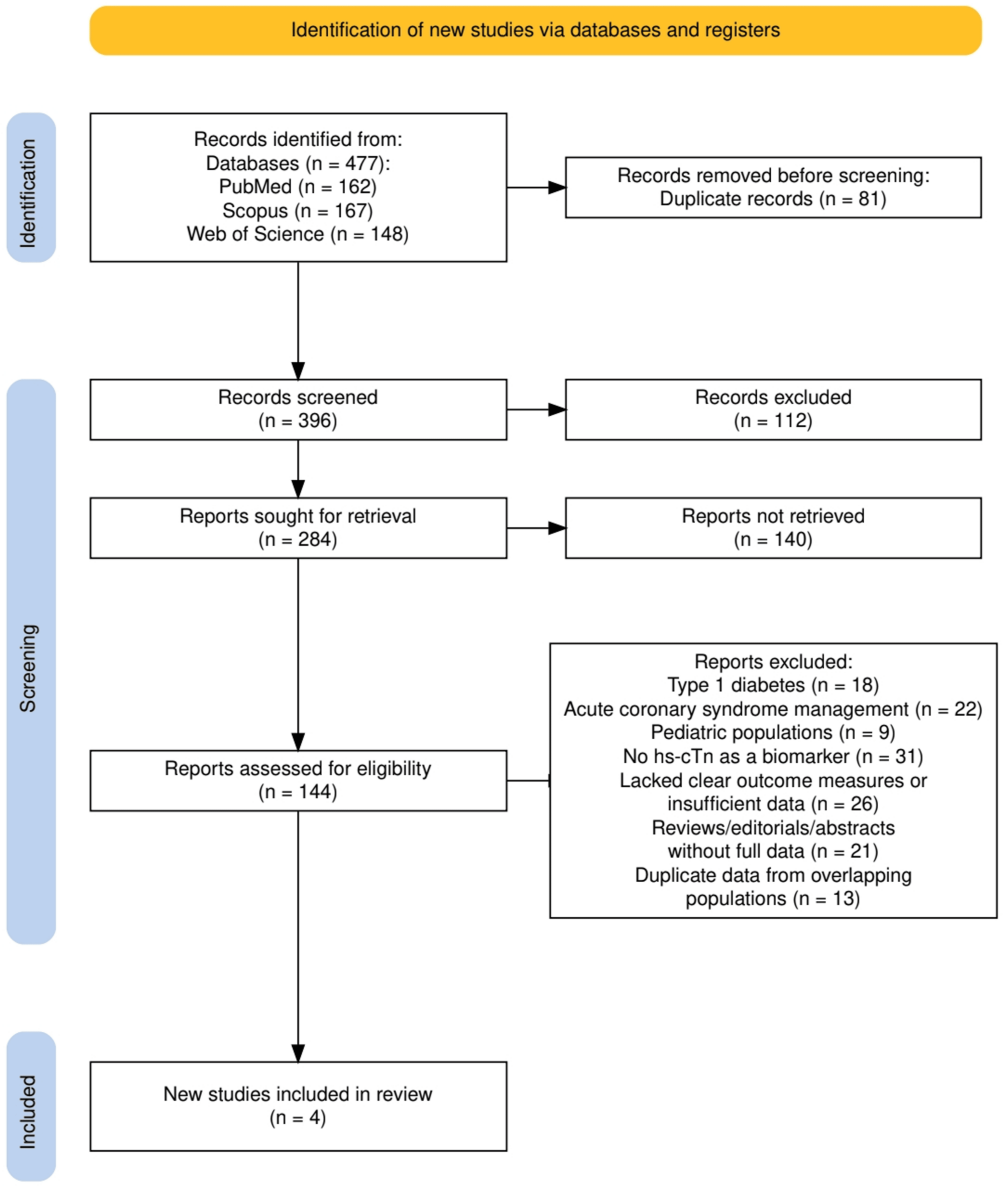
The PRISMA flowchart represents the study selection process PRISMA: Preferred Reporting Items for Systematic Reviews and Meta-Analyses

The characteristics of the selected studies are illustrated in Figure 1, which presents the PRISMA flow diagram summarizing the identification, screening, and inclusion process. A total of 477 records were initially identified across three electronic databases: PubMed (n = 162), Scopus (n = 167), and Web of Science (n = 148). After removing 81 duplicate entries, 396 records were screened, from which 112 were excluded based on title and abstract. Of the 284 full-text reports sought, 140 could not be retrieved, and 144 were assessed for eligibility. Following full-text screening, 140 studies were excluded based on predefined criteria, including focus on type 1 diabetes, acute coronary syndromes, pediatric populations, lack of hs-cTn usage, insufficient data, or duplication. Ultimately, four high-quality studies met all eligibility criteria and were included in the final qualitative synthesis. These studies varied in design but were unified by their focus on the prognostic value of high-sensitivity troponin in predicting long-term cardiovascular outcomes in patients with type 2 diabetes mellitus.

Characteristics of the Selected Studies

As detailed in Table 1, the four studies included in this review varied in design but collectively contributed valuable insights into the prognostic role of high-sensitivity cardiac troponin (hs-cTn) in patients with type 2 diabetes mellitus (T2DM). The selected studies encompassed randomized controlled trials, a post hoc analysis of an RCT, and a long-term observational cohort study. Sample sizes ranged from 669 to over 5,000 participants, and follow-up durations varied from 15 months to a median of 11 years, allowing for both intermediate and long-term outcome assessment. The populations studied included T2DM patients with stage B heart failure, post-acute coronary syndrome, diabetic nephropathy, and stable outpatient profiles. All studies employed high-sensitivity troponin assays, primarily hs-TnT and one using hs-TnI, with varying thresholds or stratification criteria. Clinical outcomes assessed included cardiovascular mortality, all-cause mortality, heart failure hospitalization, renal events, and functional decline. Despite differences in study settings and endpoints, the findings consistently demonstrated that elevated hs-cTn levels were associated with adverse clinical outcomes, reinforcing the biomarker’s potential role in long-term risk stratification in diverse T2DM populations.

**Table 1:** Summary of studies included, detailing study characteristics, population, and key findings on hs-cTn hs-cTn: High-Sensitivity Cardiac Troponin; MI: Myocardial Infarction; PPV: Positive Predictive Value; NPV: Negative Predictive Value; AUC: Area Under the Curve; CI: Confidence Interval; NT-proBNP: N-terminal pro B-type natriuretic peptide.

Study (Author, Year)	Study Design	Population	Sample Size (n)	Follow-Up Duration	hs-cTn Type & Cutoff	Outcome(s) Measured	Key Findings
Marwick et al., 2025 [10]	Phase 3 RCT (Subgroup analysis)	T2DM patients with stage B heart failure (SBHF)	1858 screened; 1463 with DbMD; 669 followed	15 months	hs-TnT ≥10 ng/L (women), ≥16 ng/L (men)	Exercise capacity (peak VO₂), grouped by phenotype	Isolated biomarker elevation present in 49%; reduced VO₂ more common in those with combined dysfunction; no significant difference in decline across groups
Sharma et al., 2020 [11]	Multicenter RCT (biomarker subanalysis)	T2DM patients after recent acute coronary syndrome	5154 with biomarker data	Median 18 months	hs-TnI (cutoff not stated in abstract)	CV death, HF hospitalization, elevated NT-proBNP, loop diuretic initiation	hs-cTnI contributed to biomarker-based risk model; NT-proBNP strongest predictor; C-statistic = 0.72
Bidadkosh et al., 2017 [12]	Post hoc analysis of RCT (Sun-MACRO)	T2DM patients with nephropathy	861	Not explicitly stated (Sun-MACRO median ~12–18 months)	hs-TnT; median 30 ng/L (IQR: 20–47 ng/L)	Renal events, cardiovascular events, mortality	hs-TnT independently predicted renal events (HR 2.09); not independently associated with CV events after NTproBNP adjustment
Hendriks et al., 2016 [13]	Observational cohort study (ZODIAC-37)	Stable outpatients with T2DM in primary care	1133	Median 11 years (IQR 7–14 years)	hs-TnT; <3 ng/L (undetectable), 3–14 ng/L (low), ≥14 ng/L (elevated)	All-cause mortality, cardiovascular mortality	hs-cTnT predicted all-cause (HR 1.30) and CV mortality (HR 1.33); strong prognostic performance (C-statistic 0.72–0.74)

Quality Assessment

As summarized in Table 2, the overall quality of the included studies was assessed using appropriate tools based on study design, namely the Cochrane Risk of Bias (RoB) 2.0 tool (www.cochrane.org) [14] for randomized trials and the Newcastle-Ottawa Scale (NOS) [15] for observational cohort studies. Two randomized trials were rated as moderate to high quality, with Sharma et al. [11] receiving a high-quality rating due to consistent low risk across all domains, while the Marwick et al. [10] study showed some concerns in detection bias due to a lack of clarity regarding blinding in subgroup analyses. The post hoc analysis by Bidadkosh et al. presented some concerns in both selection and detection bias, leading to a moderate quality rating overall [12]. The cohort study by Hendriks et al. scored 8 out of 9 stars on the NOS, reflecting a well-defined cohort, long-term follow-up, robust outcome assessment, and appropriate adjustment for confounders, thus indicating a high level of methodological quality [13]. Overall, the included studies demonstrated acceptable to high quality, supporting the credibility of the evidence synthesized in this review.

**Table 2:** Prognostic significance of hs-cTn in predicting MACE hs-cTn: High-Sensitivity Cardiac Troponin; MACE: Major Adverse Cardiovascular Events; HR: Hazard Ratio; OR: Odds Ratio; CI: Confidence Interval; ROC: Receiver Operating Characteristic; RCT: Randomized controlled trial.

Study (Author, Year)	Tool Used	Study Design	Selection Bias	Performance Bias	Detection Bias	Attrition Bias	Reporting Bias	Overall Quality Rating
Marwick et al., 2025 [10]	Cochrane RoB 2.0	RCT (Subgroup)	Low	Low	Some concerns (no mention of blinding in subgroups)	Low	Low	Moderate
Sharma et al., 2020 [11]	Cochrane RoB 2.0	RCT (Biomarker analysis)	Low	Low	Low	Low	Low	High
Bidadkosh et al., 2017 [12]	Cochrane RoB 2.0	Post hoc of RCT	Some concerns (post hoc design)	Low	Some concerns	Low	Low	Moderate
Hendriks et al., 2016 [13]	Newcastle-Ottawa Scale	Observational cohort	★★★ (well-defined cohort)	★★ (follow-up of 11 years)	★★★ (adjusted models used)	★★ (some loss to follow-up)	Not applicable	Good (8/9 stars)

Discussion

The findings from the included studies collectively highlight the growing clinical value of high-sensitivity cardiac troponin (hs-cTn) as a prognostic biomarker in patients with type 2 diabetes mellitus (T2DM). Across diverse populations and study designs, hs-cTn demonstrated consistent associations with adverse long-term outcomes. In the ARISE-HF trial, nearly half of the patients exhibited isolated elevations in hs-TnT, with reduced exercise capacity more pronounced in those with additional systolic or diastolic dysfunction, though longitudinal decline did not significantly differ between groups [10]. The EXAMINE trial provided further evidence of the utility of hs-cTnI in risk modeling following acute coronary events, where it contributed to a predictive model for heart failure and cardiovascular events, with a respectable C-statistic of 0.72 [11]. Meanwhile, the post hoc analysis from the Sun-MACRO trial by Bidadkosh et al. found that hs-TnT independently predicted renal events in T2DM patients with nephropathy, although its association with cardiovascular outcomes diminished after adjusting for NT-proBNP [12]. The most compelling long-term data came from the ZODIAC-37 cohort, which showed that higher baseline hs-TnT levels were independently associated with both all-cause and cardiovascular mortality over a median follow-up of 11 years, with strong discriminatory performance [13]. Taken together, these findings underscore the potential role of hs-cTn, particularly hs-TnT, in identifying T2DM patients at heightened risk for long-term adverse outcomes, even in the absence of overt cardiovascular disease [10-13].

The findings of this review reinforce the evolving understanding that high-sensitivity cardiac troponins, particularly hs-TnT and hs-TnI, serve as valuable indicators of subclinical myocardial injury and hold significant prognostic utility in patients with type 2 diabetes mellitus (T2DM) [16]. In contrast to traditional risk scores that often underestimate cardiovascular risk in diabetic populations, hs-cTn offers a more direct reflection of ongoing myocardial stress and damage, even in stable or asymptomatic individuals [17]. The studies reviewed demonstrate that elevated hs-cTn levels are not only common in T2DM but are also independently associated with increased risks of cardiovascular and all-cause mortality, heart failure progression, and, to some extent, renal events [18]. This aligns with current clinical knowledge recognizing T2DM as a condition of chronic vascular inflammation and myocardial vulnerability. Importantly, the predictive performance of hs-cTn appears robust across different diabetic subgroups, whether post-acute coronary syndrome, with nephropathy, or in stable outpatient settings, highlighting its potential as a universal tool for long-term cardiovascular risk stratification in diabetes care [19].

Previous literature has consistently acknowledged the elevated cardiovascular risk in patients with type 2 diabetes, yet the integration of cardiac biomarkers such as hs-cTn into routine risk prediction remains limited. Our findings are in alignment with several earlier studies that have demonstrated the prognostic relevance of hs-cTn in various clinical settings, including heart failure, chronic kidney disease, and general population cohorts [20]. However, what sets this review apart is its exclusive focus on T2DM populations, where myocardial injury may be more insidious and under-recognized. Prior systematic reviews and guidelines have emphasized N-terminal pro B-type natriuretic peptide (NT-proBNP) as a cornerstone biomarker for cardiovascular risk assessment [21], but our synthesis highlights that hs-cTn, especially when used in combination with other markers, can provide complementary and sometimes superior risk discrimination. These observations support emerging literature suggesting that even low-grade troponin elevations within the normal reference range can have significant prognostic implications in diabetics [22], a nuance that is not yet fully captured in most guideline-based risk stratification tools.

From a clinical standpoint, the incorporation of hs-cTn measurement into the routine evaluation of patients with type 2 diabetes could represent a paradigm shift in cardiovascular risk management. Its ability to detect subclinical myocardial injury offers clinicians an opportunity to identify high-risk individuals who may not yet exhibit symptoms or have overt cardiac dysfunction. In real-world practice, hs-cTn could serve as a cost-effective, minimally invasive tool to enhance existing risk prediction models and guide the intensity of preventative therapies, such as statins, angiotensin converting enzyme (ACE) inhibitors, or sodium-glucose co-transporter-2 (SGLT2) inhibitors [23]. Furthermore, periodic monitoring of hs-cTn levels could help track disease progression or therapeutic response, especially in patients with coexisting nephropathy or previous cardiovascular events [24]. As the evidence base grows, the integration of hs-cTn into clinical decision-making algorithms may help personalize care, improve outcomes, and reduce the burden of cardiovascular complications in the diabetic population.

This systematic review benefits from several methodological strengths that enhance the reliability and clinical relevance of its findings. The inclusion criteria were deliberately focused to capture studies involving patients with type 2 diabetes mellitus and explicitly evaluating the prognostic role of high-sensitivity cardiac troponin in relation to long-term cardiovascular outcomes. By selecting studies with clearly defined endpoints, validated biomarker assays, and robust follow-up durations, ranging from over a year to more than a decade, this review provides a comprehensive and time-relevant synthesis of evidence. The use of standardized tools for quality assessment, including the Cochrane Risk of Bias tool and the Newcastle-Ottawa Scale, further strengthens the appraisal of included literature, ensuring consistency in evaluating methodological rigor and reducing bias.

Nonetheless, certain limitations must be acknowledged. The relatively small number of eligible studies and their heterogeneity in design, population characteristics, and outcome definitions may introduce variability that limits direct comparison and meta-analysis. Some studies lacked detailed reporting on hs-cTn assay types, cutoffs, or stratification strategies, which may affect the reproducibility of findings in clinical settings. Furthermore, the post hoc nature of biomarker analyses in some trials and the absence of individual patient data restrict the ability to perform deeper subgroup or interaction analyses. Generalizability may also be limited, as most studies focused on older populations or those with existing comorbidities, potentially underrepresenting younger or newly diagnosed T2DM patients.

Future research should aim to build upon the current evidence by conducting large-scale, prospective cohort studies specifically designed to evaluate high-sensitivity cardiac troponin as a risk stratification tool in type 2 diabetes mellitus. There is a clear need for standardized hs-cTn assay protocols and clinically meaningful thresholds tailored to diabetic populations, accounting for sex, age, renal function, and baseline cardiovascular risk. Additionally, future studies should explore the utility of serial troponin measurements over time to assess dynamic risk and response to interventions. Integrating hs-cTn into multi-marker models alongside other emerging biomarkers such as NT-proBNP and growth/differentiation factor 15 (GDF-15) may also enhance predictive accuracy [12]. Importantly, randomized trials assessing whether biomarker-guided therapeutic strategies lead to improved cardiovascular outcomes in diabetics would be instrumental in translating this biomarker from prognostic relevance to actionable clinical utility. At the same time, practical challenges, such as assay availability, cost-effectiveness, variability in global healthcare access, and the psychological impact of detecting asymptomatic troponin elevations, must be addressed. These factors underscore the importance of validating hs-cTn-based models across multi-ethnic cohorts and diverse healthcare systems to ensure both clinical and contextual applicability.

## Conclusions

This systematic review highlights the robust prognostic utility of high-sensitivity cardiac troponin in predicting long-term cardiovascular outcomes in patients with type 2 diabetes mellitus. Elevated hs-cTn levels-particularly hs-TnT-were consistently linked to increased risks of cardiovascular and all-cause mortality, heart failure progression, and renal complications, even among asymptomatic individuals without overt cardiovascular disease. These findings position hs-cTn as a sensitive and clinically meaningful biomarker of subclinical myocardial injury, offering added value beyond traditional risk models. Despite its underuse in routine diabetic care, integrating hs-cTn into cardiovascular risk assessment could enable earlier identification of high-risk individuals and support more tailored, preventive strategies. As evidence accumulates, hs-cTn testing may become a cornerstone of personalized cardiovascular care in this high-risk population, improving long-term outcomes and guiding more effective clinical decision-making.
